# Impact on Life Expectancy of Withdrawing Thiopurines in Patients with Crohn’s Disease in Sustained Clinical Remission: A Lifetime Risk-Benefit Analysis

**DOI:** 10.1371/journal.pone.0157191

**Published:** 2016-06-06

**Authors:** Julien Kirchgesner, Laurent Beaugerie, Fabrice Carrat, Harry Sokol, Jacques Cosnes, Michaël Schwarzinger, BERENICE Study Group

**Affiliations:** 1 Department of Gastroenterology, AP-HP, Hôpital Saint-Antoine, Paris, France; 2 UMR-S 1136, INSERM & UPMC Univ Paris 06, Paris, France; 3 ERL 1057 INSERM/UMRS 7203 and GRC-UPMC 03, UPMC Univ Paris 06, Paris, France; 4 Department of Public Health, AP-HP, Hôpital Saint-Antoine, Paris, France; 5 THEN, Translational Health Economics Network, Paris, France; 6 UMR 1137, INSERM, Infection, Antimicrobials, Modelization, Evolution (IAME), Paris, France; University Hospital Llandough, UNITED KINGDOM

## Abstract

**Objective:**

Long-term treatment with thiopurines is associated with a decreased risk of Crohn’s disease (CD) flare but an increased risk of various cancers depending on gender, age, and presence of extensive colitis. We evaluated risks and benefits of withdrawing thiopurines in patients with CD in prolonged remission.

**Methods:**

We developed a Markov model assessing risks and benefits of withdrawing thiopurines compared to continuing thiopurines in a lifetime horizon. The model was stratified by age (35 and 65 years old at thiopurine withdrawal), gender and presence of extensive colitis. Parameter estimates were taken from French cohorts and hospital databases, cancer and death national registries and published literature. Life expectancy, rates of relapse, serious adverse events, and causes-of-death were evaluated.

**Results:**

In patients without extensive colitis, continuing thiopurines increased life expectancy up to 0.03 years for 35 year-old men and women but decreased life expectancy down to 0.07 years for 65 year-old men and women. Withdrawal strategy became the preferred strategy at 40.6 years for men, and 45.7 years for women without extensive colitis. In patients with extensive colitis, continuation strategy was the preferred strategy regardless of age. Risk-benefit analysis was not modified by duration of CD activity.

**Conclusions:**

Factors determining life expectancy associated with withdrawal or continuation of thiopurines in patients with CD and in sustained clinical remission vary substantially according to gender, age and presence of extensive colitis. Individual decisions to continue or withdraw thiopurines in patients with CD in sustained remission should take into account these parameters.

## Introduction

Crohn’s disease (CD) is a chronic idiopathic inflammatory bowel disease with relapsing and remitting episodes that may lead to irreversible intestinal lesions, severe disability, and excess mortality.[1–3] Thiopurines include azathioprine and its metabolite 6-mercaptopurine. These two immunosuppressive drugs (thiopurines) have been shown to be superior to placebo for inducing and maintaining clinical remission of CD: about five CD patients need to be continuously treated with thiopurines to prevent one relapsing episode.[4] Thiopurines are currently recommended as first-line maintenance therapy in various clinical situations within the first year of CD onset,[5] and the prevalence of CD patients exposed to prolonged immunosuppressive treatment is increasing, e.g., about 40% in France in 2006.[6]

Prolonged treatment with thiopurines may be associated with excess mortality risks due to opportunistic viral infections[7,8] and lymphoma.[6] In addition, second-line maintenance therapy with tumor necrosis factors inhibitors (anti-TNFs) is associated with excess mortality risks of prolonged immunosuppressive treatment.[9] In a recent survey, about 60% of patients on maintenance therapy reported that they were concerned by serious adverse events (SAE) and engaged intentionally in a non-adherent behavior,[10] whereas another recent survey conclude that patient may accept high risk levels of lymphoma and serious infection to maintain disease remission.[11] Risk-benefit assessment of medications is therefore strongly needed to provide relevant information to patients.

In the present study, we developed a model-based risk-benefit analysis of withdrawing thiopurines in CD patients in prolonged remission. The model makes explicit the trade-off between two excess mortality risks regarding life expectancy: 1) withdrawing thiopurines increases the cumulative rate of severe relapse over time as compared to continuing thiopurines; 2) continuing thiopurines increases the risks of serious adverse events including a sharp increase of cancer-related risks with age and serious infections. Because of two main characteristics of CD patients regarding excess mortality risks, we conducted threshold analyses on age stratified by gender and presence of extensive colitis.[12] Finally, extensive sensitivity analyses were performed.

## Materials and Methods

We developed a decision-analytic Markov model that follows cohorts of CD patients in prolonged remission stratified by age, gender and presence of extensive colitis (as defined by a proportion of the colonic mucosal area macroscopically or microscopically affected by disease>50%).[12] We used the model to identify the lifetime risks and benefits of withdrawing thiopurines, providing practical insights relevant for the management of CD.

### Decision Tree and Markov Model

**Fig 1:**
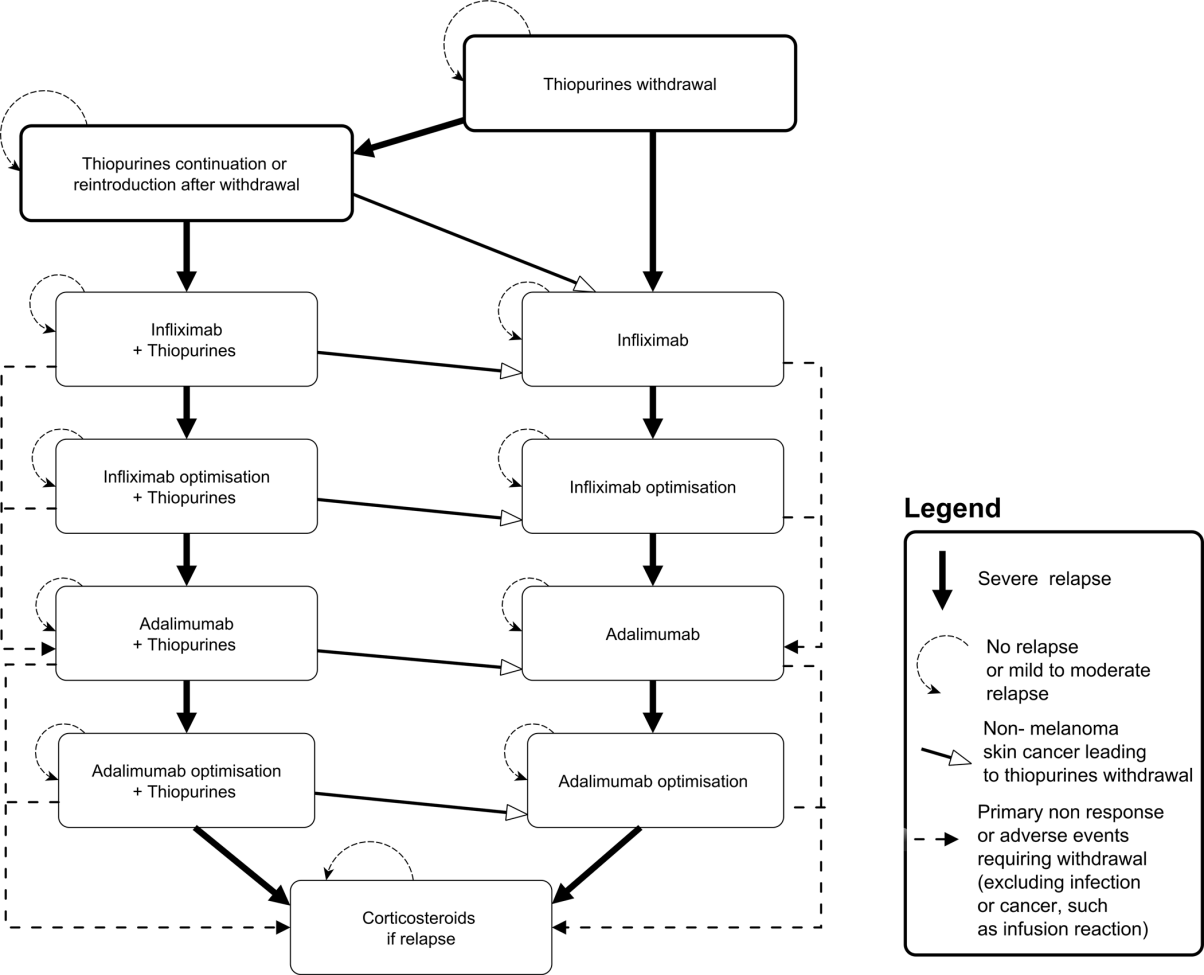
Events related to Crohn’s disease and treatment and associated with mortality risk.

The Markov model simulates the natural history of CD with relapsing and remitting episodes (Fig 1). The target population is initially under thiopurines since the first year of CD onset and set in prolonged remission since four years with thiopurines, for a total of 5 years under thiopurines. In the base-case scenario, we assumed that chronic bowel inflammation will remain active for 15 years after cohort entry and incur a baseline risk of relapse every year. Patients remaining in remission or developing a mild relapse continue on the same maintenance therapy at the next Markov cycle. Patients developing a severe relapse are hospitalized with an excess mortality risk depending on the need for surgery. In case of intestinal resection, we assumed that the baseline risk of relapse decreases for two years.[13] In all patients surviving severe relapse, maintenance therapy is changed at the next Markov cycle. After 15 years, patients are no longer at risk of relapse and resume to the life expectancy of the general population. We assumed that maintenance therapy is stopped in patients without CD activity.

**Fig 2:**
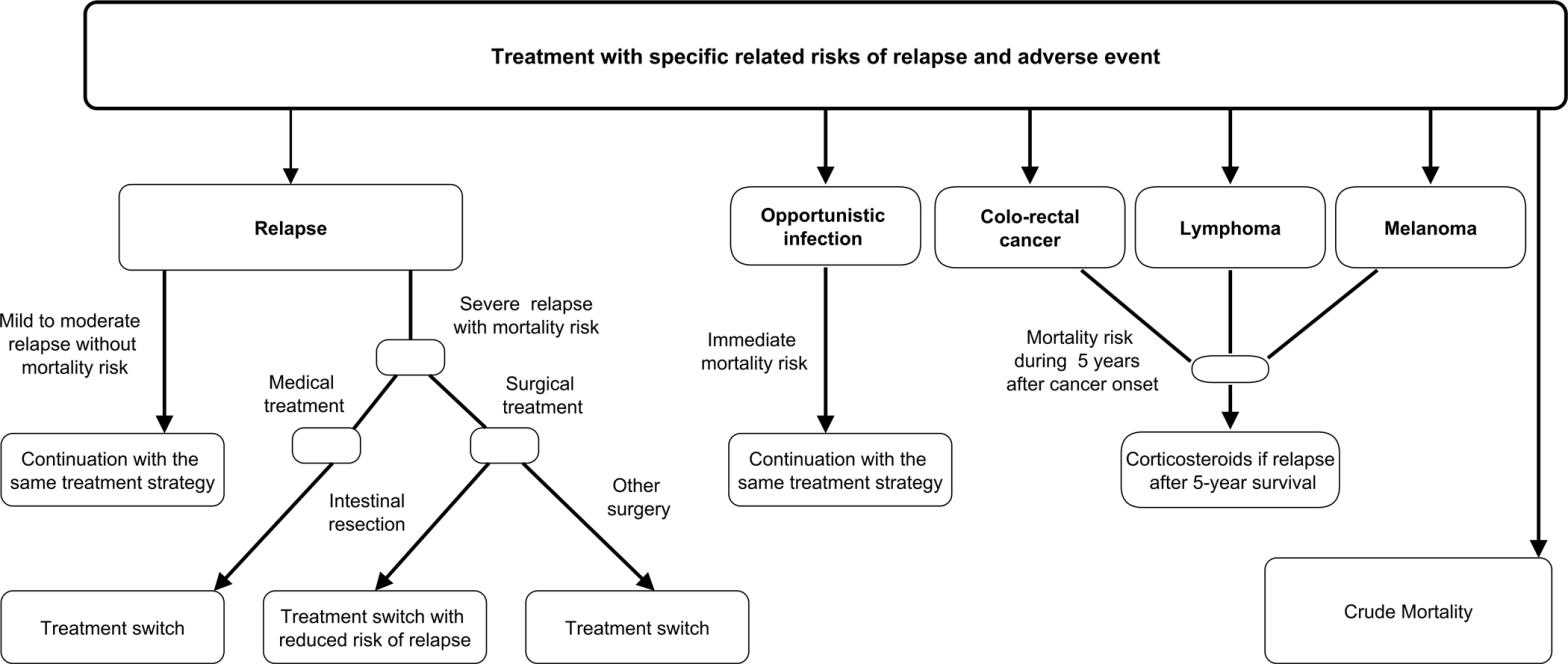
Overview of the Decision Model: therapeutic management.

We compared two alternative strategies: withdrawing (strategy W) versus continuing (strategy C) maintenance therapy with thiopurines (Fig 2). A clinical trial did not show the noninferiority of withdrawing compared to continuing maintenance therapy regarding the relapse rate at 18 months,[14] and the relapse rate became significantly higher at 5 years.[15] We assumed conservatively that strategy W increases the baseline risk of relapse from the first Markov cycle onwards. Patients who relapse after discontinuation of thiopurines are treated with a second course of thiopurines, and we assumed that remission is achieved in all patients who previously respond to thiopurines.[15] However, we assumed conservatively that those patients back on maintenance therapy with thiopurines would not resume to the baseline risk of relapse of strategy C.

Once on maintenance therapy with thiopurines, all patients of strategies C and W receive the same standard of care over time depending on the occurrence of a severe relapse or SAE. Severe relapse is followed at the next Markov cycle by a gradual response with several lines of maintenance therapy involving Anti-TNFs. We assumed that no other Anti-TNFs than infliximab and adalimumab would be available, and only steroids would be administered in patients having failed all lines of maintenance therapy. The doses of each Anti-TNFs are systematically intensified before discontinuation, except if treatment failed at induction therapy (i.e., primary non-response) or SAE occurred.

In the base-case scenario, we considered 4 SAE as defined by an excess mortality risk (Fig 1). Patients exposed to all immunosuppressive drugs may develop severe opportunistic infections: immunosuppressive drugs are temporally stopped for six months on average and then resumed. Patients exposed to immunosuppressive drugs are at increased risk of lymphoma (thiopurines) and melanoma skin cancer (Anti-TNFs), but at decreased risk of colorectal cancer (CRC) in presence of extensive colitis (thiopurines). In patients diagnosed with cancer, we assumed that patients were mostly at risk of specific mortality from cancer for 5 years. Maintenance therapy was discontinued in all cancer patients, but steroids may be used in cancer survivors resuming to CD activity and relapsing. Lastly, patients under thiopurines may present non melanoma skin cancer (non-MSC) leading to withdrawal of thiopurines and monotherapy of Anti-TNFs if required. In sensitivity analyses, we introduced other SAE recently attributed to thiopurines: acute myeloid leukemia;[16] urinary tract cancer;[17] and bone marrow suppression.[18]

The model was implemented in TreeAge Pro 2011 (Williamstown, MA).

### Data and Sources

We estimated model parameters from French hospital Diagnosis Related Groups (DRGs) database (Programme de médicalisation des systèmes d’information (PMSI)),[19] the French cancer and death national registries,[20–24] the CESAME cohort,[25] the Saint-Antoine hospital IBD registry (MICISTA registry), and from the literature otherwise [4,14,26–42] (Table 1). The study was approved by the French National Commission for Data Protection (CNIL DE-2013-068). The requirement for informed consent was waived because the study used de-identified data.

**Table 1:** Model parameter values.

Model parameter			Base	Min	Max	Source
*Target population characteristics*						
	Female (%)		60	-	-	
	Cohort starting age, years		35	25	65	
	Duration of Crohn's disease activity, years		20	20	55	
	Extensive colitis (%)		39	-	-	25
*Annual risk of relapse*						
	*Before intestinal resection*					
		Thiopurines continued	0.093	0.015	0.170	14, 26
		Relative Risk of relapse when withdrawing thiopurines versus continuing	2.793	2.185	9.549	14, 26
		Thiopurines resumed after withdrawal	0.358	0.243	0.473	4
		Thiopurines and Infliximab	0.348	0.291	0.405	27
		Infliximab monotherapy	0.537	0.483	0.592	27
		Infliximab optimized and thiopurines	0.518	0.425	0.610	28
		Infliximab optimized monotherapy	0.532	0.389	0.675	28
		Primary non-response to Infliximab	0.092	0.066	0.118	30
		Thiopurines and Adalimumab	0.570	0.468	0.673	30
		Adalimumab monotherapy	0.574	0.530	0.619	30
		Adalimumab after optimization (with or without thiopurines)	0.528	0.365	0.691	31, Expert opinion
		Primary non-response to Adalimumab	0.041	0.023	0.060	32
		Steroids only for induction of remission	0.402	0.318	0.485	33
	*After intestinal resection*					
		Thiopurines	0.157	0.106	0.207	34
		Infliximab (without or with thiopurines)	0.01	0	0.05	35
		Adalimumab (without or with thiopurines)	0.065	0	0.185	36
*Relapse-related events*						
	Severe relapse		0.290	0.20	0.38	MICISTA Cohort
	Surgery for severe relapse		0.379	0.20	0.56	MICISTA Cohort
	Intestinal resection for surgery		0.724	0.50	1.00	37
	Duration of decreased risk of relapse after intestinal resection (years)		2	1	5	Expert opinion
*Annual risk of Serious Adverse Events*						
	Neutropenia with thiopurines		0.0015	0.0012	0.0019	French DRG 2008–09
	Opportunistic (viral) infection with thiopurines		0.0017	0.0013	0.0022	French DRG 2008–09
	Opportunistic infection with anti-TNF drugs		0.0113	0.0090	0.0139	French DRG 2008–09
	SAE due to infliximab (opportunistic infections and cancers excluded)		0.018	0.013	0.022	38
	SAE due to adalimumab (opportunistic infections and cancers excluded)		0.001	0	0.003	39
	Standardized Incidence Ratio of Lymphoma					
		Thiopurines continuation	4.92	3.10	7.78	40
	Standardized Incidence Ratio of Colorectal cancer					
		Longstanding colitis	9.04	4.81	15.5	25
		Longstanding colitis, Thiopurines continuation	0.28	0.09	0.89	25
	Standardized Incidence Ratio of melanoma skin cancer (MSC)					
		Anti-TNF continuation	1.37	1.10	1.70	40
	Standardized Incidence Ratio of non-melanoma skin cancer (non-MSC)					
		Thiopurines continuation	2.28	1.50	3.45	42
	Standardized Incidence Ratio of acute myeloid leukemia					
		Thiopurines continuation	6.98	1.44	20.36	16
	Standardized Incidence Ratio of urinary tract cancer					
		Thiopurines continuation	3.40	1.47	6.71	17
*Mortality rates of Severe Adverse Events*						
	Neutropenia with thiopurines		0.0328	0.0040	0.1135	French DRG 2008–09
	Opportunistic infection with thiopurines		0.0286	0.0035	0.0994	French DRG 2008–09
	Opportunistic infection with anti-TNF drugs		0.0460	0.0127	0.1136	French DRG 2008–09
	Age-adjusted mortality rate of severe flare without surgery		0.0021	0.0011	0.0036	French DRG 2008–09
	Age-adjusted mortality rate of severe flare with surgery		0.0103	0.0056	0.0177	French DRG 2008–09

Min and Max were computed from exact 95% confidence intervals of percentages.

#### Target population characteristics

Baseline characteristics of the overall population of CD patients in prolonged remission were estimated from the French National Hospital DRG database (PMSI). About 34,730 adult patients were hospitalized with a primary diagnosis of CD in 2008–2009 (ICD-10 medical code: K50). The proportion of female was 60%, and age had a lognormal distribution with a mean (median) age of 40 (38) years old. The proportion of CD patients with extensive colitis (39.0%) was estimated from the CESAME cohort.[25]

#### Natural history of CD

The annual risk of relapse on maintenance therapy was estimated from clinical trials or large cohorts of CD patients (Table 1). The relative risk of relapse of withdrawing thiopurines was estimated from two clinical trials conducted in CD patients in prolonged remission for more than 2[26] and 3.5[14] years. The proportion of patients hospitalized for a severe relapse (29.0%), and among them, the proportion of patients undergoing surgery (37.9%) were estimated from 357 CD patients having received thiopurines for at least 3 years with a complete follow-up in the Saint-Antoine hospital CD registry (MICISTA). The proportion of patients undergoing surgery and having an intestinal resection (72.4%) was estimated from the Olmsted Cohort Study.[37]

Excess mortality risks due to severe relapse were estimated from the French National Hospital DRG database (PMSI). Among CD patients aged 25 to 80 years old, 42,264 hospital stays for a severe relapse were selected in 2008–2009. In-hospital mortality rates were significantly increased with surgery and an older age, but not gender.

#### Serious adverse events of prolonged immunosuppressive treatment

The frequency of opportunistic infections was measured from the French National Hospital DRG database (PMSI). Among CD patients aged 25 to 80 years old without cancer or HIV infection, 70 primary diagnoses of opportunistic viral infection were attributed to thiopurines in the absence of hospital records of Anti-TNFs (3 patients died: 1 herpes simplex virus, 1 cytomegalovirus, 1 varicella-zoster virus), and 87 primary diagnoses of opportunistic infection were otherwise attributed to Anti-TNFs (4 patients died: 1 aspergillosis, 1 candidemia, 1 pneumocystis, 1 cytomegalovirus). Drug exposure to Anti-TNFs was directly measured from hospital records in 2008–2009 (7,709 patient-years) and indirectly estimated for thiopurines with use of the proportion of CD patients treated in the CESAME cohort (40,579 patient-years). Other risks of SAE due to Anti-TNFs and requiring drugs discontinuation were estimated from the literature.[38,39]

The annual risk of cancer was calculated as the product of the age- and gender-specific incidence rate observed in French cancer registries (S1 Table),[20,21] and the standardized incidence ratio (SIR) with immunosuppressant drug exposure. We obtained SIR according to our predefined sequence for use of drugs and drug combinations from meta-analyses (Table 1).[25,40–42] In case of lymphoma occurrence, we assumed that 90% of patients develop non-Hodgkin’s lymphoma (NHL) of higher age-specific incidence and mortality rates as compared to Hodgkin’s lymphoma. Five-year relative mortality rates were obtained by age and gender from the French cancer registries (S2 Table).[22,23]

Cohorts of CD patients were further stratified by the presence of extensive colitis. In a recent study, CD patients with extensive colitis for more than 10 years had a higher risk of colorectal cancer than other CD patients, but maintenance therapy with thiopurines was found protective.[25] CD patients without extensive colitis have the same risk of colorectal cancer than the general population.[25,43]

#### Background (competing) mortality

Competing mortality was derived from 2010 French life tables after exclusion of all causes-of-death explored in the model (S3 Table).

### Risk-Benefit Analysis

The primary outcome of the risk-benefit analysis was life expectancy. Secondary outcomes included the probability of severe relapse with and without surgery, opportunistic infection, lymphoma, colorectal cancer, melanoma skin cancer, and causes-of-deaths. Probabilities were calculated over the duration of CD activity for CD-related events and over lifetime otherwise. In addition, we measured quality-adjusted life years (QALYs) with use of published utility estimates for each health state of the model (S4 Table).

In the first step of the risk-benefit analysis, we conducted deterministic analyses for patients aged 35 and 65 years old at the decision, stratified by gender and presence of extensive colitis. 35 years old correspond to 5 years after the mean age of CD onset [3] and 65 years old is a classic threshold for increase of SAE. Then, we conducted threshold analyses on age to assess when strategy W decreased life expectancy.

Secondly, the robustness of study results was assessed by relaxing key assumptions of the model regarding: 1) better effectiveness of strategy W (i.e., at withdrawal and when thiopurines are resumed; 2) lower protective effect of thiopurines on colorectal cancer and lower relative risk of colorectal cancer in case of extensive colitis); 3) the addition of other SAE (myelotoxicity, acute myeloid leukemia, urinary tract cancer); 4) longer duration of CD activity.

Finally, we performed a probabilistic sensitivity analysis to assess to what extent withdrawing thiopurines may be an acceptable strategy overall. We conducted Monte Carlo simulations of 10, 000 CD cohorts incorporating all uncertainties from variables distributions derived from the literature (S5 Table) and measured how frequently strategy W decreased life expectancy as compared to strategy C.

## Results

### Stratified deterministic analyses

**Fig 3:**
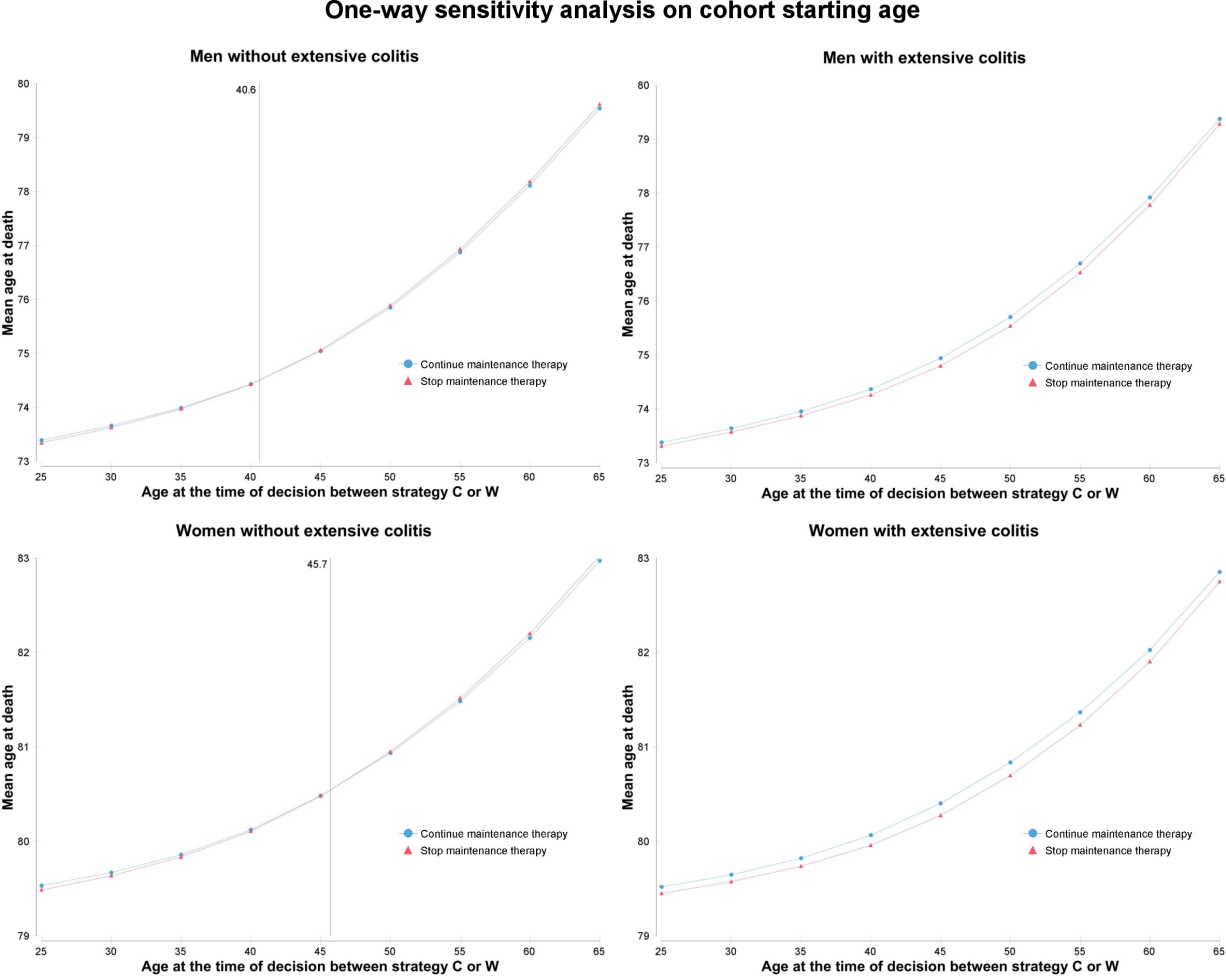
One-way sensitivity analysis on cohort starting age. (Mean age increase at death associated to age increase at the time of decision is related to shorter exposition to competing mortality risk)

Life expectancy and causes of death from continuing or withdrawing maintenance therapy with thiopurines in cohorts without extensive colitis are provided in Table 2. In 35 year-old patients without extensive colitis, continuing thiopurines increased life expectancy of all patients in prolonged remission. In 35 year-old men without extensive colitis, continuing thiopurines reduced deaths related to severe relapse by half (0.31% versus 0.64%), although deaths related to SAE increased by 17%. Similar results were found in 35 year-old women without extensive colitis. In 65 year-old patients without extensive colitis, withdrawing thiopurines increased life expectancy for men and women. As compared to 35 year-old patients, decision reversal was related to lower rates of death caused by severe relapse and higher rates of death caused by SAE. In CD patients in prolonged remission without extensive colitis, withdrawing thiopurines increased life expectancy after 40.6 years in men and 45.7 years in women (Fig 3).

**Table 2:** Life expectancy and causes-of-death from continuing (C) or withdrawing (W) maintenance therapy with thiopurines in stratified CD cohorts without extensive colitis.

		Male, 35y., CD still active for 15y.		Male, 65y., CD still active for 15y.		Female, 35y., CD still active for 15y.		Female, 65y., CD still active for 15y.	
		C	W	C	W	C	W	C	W
Life expectancy, age at death		73.99	73.97	79.54	79.61	79.86	79.83	82.97	83.02
Loss in life expectancy as compared to general population, years		-0.25	-0.27	-0.18	-0.11	-0.28	-0.31	-0.17	-0.12
Causes of death, %									
	Severe relapse without surgery	0.08%	0.16%	0.07%	0.12%	0.08%	0.16%	0.07%	0.14%
	Severe relapse with surgery	0.23%	0.48%	0.21%	0.37%	0.24%	0.49%	0.22%	0.42%
	Opportunistic infection	0.18%	0.12%	0.17%	0.09%	0.19%	0.12%	0.18%	0.10%
	Lymphoma	0.76%	0.67%	1.81%	1.01%	0.53%	0.48%	1.28%	0.72%
	Colorectal cancer	1.90%	1.91%	1.61%	1.63%	1.49%	1.50%	1.21%	1.22%
	Melanoma skin cancer	0.57%	0.21%	0.31%	0.17%	0.63%	0.18%	0.24%	0.12%
	Other causes of death	96.28%	96.45%	95.82%	96.61%	96.84%	97.07%	96.80%	97.28%
	Relapse	0.31%	0.64%	0.28%	0.49%	0.32%	0.65%	0.29%	0.56%
	SAE	3.41%	2.91%	3.90%	2.90%	2.84%	2.28%	2.91%	2.16%

Events rates associated with continuing or withdrawing thiopurines are shown in Table 3. In 35 year-old men without extensive colitis, continuing thiopurines increased lifetime risks for lymphoma by 18% and decreased lifetime risks for severe flare by 52%. In 65 year-old men without extensive colitis, continuing thiopurines increased lifetime risks for lymphoma by 77%. Similar results were found in women.

**Table 3:** Events associated with continuing (C) or withdrawing (W) from maintenance therapy with thiopurines in patients without extensive colitis.

			Male, 35y., CD still active for 15y.		Male, 65y., CD still active for 15y.		Female, 35y., CD still active for 15y.		Female, 65y., CD still active for 15y.	
			C	W	C	W	C	W	C	W
Events for 1,000 patients-years										
		Severe relapse (during CD activity)	40.64	84.99	46.05	80.68	41.15	85.25	43.85	82.67
		Lymphoma	0.58	0.49	0.99	0.56	0.36	0.31	0.63	0.36
		Colorectal cancer	1.47	1.48	1.10	1.11	1.02	1.03	0.76	0.77
		Opportunistic infection (during drug exposure)	3.31	1.93	3.63	1.71	3.35	1.95	3.50	1.81
Relative risk between continuation and withdrawal strategy										
	Severe relapse		0.48		0.57		0.48		0.53	
	Lymphoma		1.18		1.77		1.16		1.77	
	Colorectal cancer		1.00		0.99		0.99		0.99	
	Opportunistic infection		1.72		2.12		1.72		1.93	
Relative risk compared to general population										
	Lymphoma		1.39	1.18	3.44	1.94	1.35	1.16	3.45	1.95
	Colorectal cancer		0.99	1.00	0.98	0.99	0.99	1.00	0.99	0.99

Life expectancy and causes of death from continuing or withdrawing maintenance therapy with thiopurines in cohorts with extensive colitis are provided in S6 Table. In 35 year-old patients with extensive colitis, continuing thiopurines increased life expectancy for men and women. In contrast to 65 year-old patients without extensive colitis, continuing thiopurines still increased life expectancy for 65 year-old men and women with extensive colitis. Continuing thiopurines remained the preferred strategy in CD patients with extensive colitis regardless of age (Fig 3). Events rates associated with continuing or withdrawing in cohorts with extensive colitis are shown in S7 Table. In 35 year-old men with extensive colitis, continuing thiopurines decreased lifetime risks for colorectal cancer by 10%. In 65 year-old men with extensive colitis, continuing thiopurines decreased lifetime risks for colorectal cancer by 44%. Similar results were found in women.

### Sensitivity analysis

The study results were sensitive to a better effectiveness of thiopurines withdrawal as compared to thiopurines continuation in patients without extensive colitis. If withdrawing thiopurines increased the relative risk of relapse below a threshold of 2.2, (base-case 2.79), withdrawing thiopurines became the preferred strategy regardless of age (S1 Fig).

The base-case model was robust to variation up to the 95% confidence bounds of relative risk of CRC in case of extensive colitis and hazard ratio for CRC between patients under thiopurines and patients not receiving thiopurines (respectively 0.89 and 4.81). In a 3-way sensitivity analysis including these values, continuing thiopurines remained the preferred strategy in 35 year-old patients with extensive colitis. If we considered the relative risk of CRC in case of extensive colitis at its lower limit of the 95% confidence interval (4.81, base-case 9.04) and the base-case hazard ratio for CRC between patients under thiopurines and patients not receiving thiopurines, withdrawal strategy remained the preferred strategy in 65 year-old patients with extensive colitis.

If QALYs based on published utility estimates were considered instead of life expectancy, continuing thiopurines was the preferred strategy, regardless of gender, age, and presence of extensive colitis (S6 Table). Complementary sensitivity analyses are detailed in supplementary material (S1 Text).

### Probabilistic analysis: simulation of 10, 000 CD cohorts

In the base-case scenario, continuing thiopurines increased life expectancy in 52.2% and 93.7% of 10, 000 simulated cohorts of CD patients in prolonged remission respectively without and with EC (Table 4); overall, continuing thiopurines incurred a gain of years as compared to thiopurines withdrawal in CD patients without and with EC of 0.03 (CI 95%, -0.03; 0.14) and 0.19 (CI 95%, 0.06; 0.24), respectively.

**Table 4:** Life expectancy from continuing (C) or withdrawing (W) maintenance therapy with thiopurines in 10,000 simulations of CD cohorts in prolonged remission.

	CD patients without extensive colitis						CD patients with extensive colitis					
	C	95% CI		W	95% CI		C	95% CI		W	95% CI	
Life expectancy, age at death, years	77.80	72.91	83.68	77.77	72.77	83.71	77.54	72.53	83.55	77.35	72.29	83.49
Loss in life expectancy as compared to general population, years	-0.47	-0.66	-0.13	-0.50	-0.80	-0.10	-0.74	-1.04	-0.26	-0.93	-1.28	-0.32
Proportion of preferred strategy	52.2%			47.8%			93.7%			6.3%		

## Discussion

Our decision analytic model demonstrates that the impact on life expectancy of withdrawing maintenance therapy with thiopurines in CD patients with prolonged remission depends on gender, age, and presence of extensive colitis. It relates primarily on the increased risks of cancer with prolonged immunosuppressive treatment by age and gender.[22,23] In this era of early and more aggressive treatment strategy, it remains important to study the impact of treatment withdrawal due to possible safety concerns and drug costs constraints.[44] CD phenotype was also a determinant characteristic in the decision since the impact of thiopurines therapy differs in patients with or without extensive colitis. The chemopreventive effect of thiopurines on colorectal cancer remains controversial. A recent meta-analysis did not find a chemopreventive effect of thiopurines on colorectal cancer in patients with IBD,[43] but a chemopreventive effect was suggested in presence of extensive colitis and IBD duration longer than 10 years.[25] Similar findings were also described in a recent Spanish cohort.[45]

The originality of our study relies on the development of a decision model depicting real-life treatment of CD. We included in the same model several treatment options that could influence the risk of relapse: primary non response of Anti-TNFs; dose adjustment before switching to another biological regimen; and different surgical procedures. One of the strengths of our model is the source of the parameters. The relapse related outcomes and risks of opportunistic infections were estimated from a large prospective cohort of CD patients (MICISTA) and the French National Hospital DRG (PMSI) database with a total of 42,264 patients identified with CD.

In the base-case analysis, the difference in life expectancy was expectedly low because competing mortality was included in the model and the two strategies only differed by their first treatment level. Patients received the same standard of care over time once on maintenance therapy with thiopurines, whatever strategy C or W. Furthermore, we made conservative assumptions as we assumed that patients under a second course of thiopurines in strategy W would not resume to the baseline risk of relapse of strategy C. This assumption favors strategy C and explains much higher rates of relapse over lifetime in strategy W. Conversely, it strengthens our results on the lifetime benefits of withdrawing thiopurines for a subset of patients in clinical remission.

Our study has several limitations. We relied on life expectancy as the primary health outcome. Accordingly, events without increased mortality risk were not taken into account in the decision-analysis model. Alternatively, the “utility” associated with health states may have been introduced to estimate gains in Quality-Adjusted Life Years (QALYs). However, we found various utility estimates in the field of inflammatory bowel disease,[46] and preferred to rely on life expectancy as primary health outcome.

The minor differences in life expectancy reported here were not clinically significant (between 7 and 36 days according to base-case scenarios). However, we observed marked differences in individual risks (related to CD activity and SAE) between therapeutic strategies according to patient characteristics and IBD phenotype. These personalized data about risks are of major importance when physicians inform their patients in a shared decision-making about treatment continuation.

We assumed an optimal adherence rate assuming that model parameters estimated from cohort studies incorporate real-life adherence. Furthermore, we considered clinical relapse without considering biological parameters such as C-reactive protein (CRP) or fecal calprotectin, because studies used for tabulating model parameters relied on the clinical assessment of relapse. Future models should include parameters from studies assessing relapse on clinical and biological parameters. Lastly, the sequential medical options included two biological agents. Future models should be updated according future approved drugs.

In conclusion, this study shows that withdrawing thiopurines is the best strategy for a large subset of patients in a simulated cohort of CD patients in clinical sustained remission regarding benefits and associated risks related to immunosuppressant drugs. This decision depends on gender, age, and disease location, as continuing thiopurines remains the best decision in case of extensive colitis regardless age and gender. The decision on continuing or withdrawing thiopurines for patients in sustained clinical remission should be tailored to these criteria.

## Supporting Information

S1 Fig Two-way sensitivity analysis of relative risk of relapse when withdrawing thiopurines versus continuing and cohort starting age. (TIFF)

S2 Fig Two-way sensitivity analysis on duration of CD activity and cohort starting age. (TIFF)

S3 Fig Two-way sensitivity analysis on cohort starting age and duration of remission during a second course of thiopurines before return to the baseline risk of relapse of strategy C. (TIFF)

S1 Table Age- and gender-specific incidence rate of lymphoma, colorectal cancer, melanoma skin cancer, acute myeloid leukemia and bladder cancer, per 100,000 (2012 French National Cancer Registry). (DOC)

S2 Table Five-year relative mortality rates of cancer (2012 French National Cancer Registry). (DOC)

S3 Table Age- and gender-specific mortality rate from other causes, per 100,000 (2010 French life tables). (DOC)

S4 Table Model utility values. (DOC)

S5 Table Model parameter distribution. (DOC)

S6 Table Life expectancy and causes of death associated with continuing (C) or withdrawing (W) from maintenance therapy with thiopurines in stratified cohorts. (DOC)

S7 Table Events associated with continuing (C) or withdrawing (W) from maintenance therapy with thiopurines. (DOC)

S1 Text Supplementary Material. (DOC)
