# Supplementary figures and images for: Impact on Life Expectancy of Withdrawing Thiopurines in Patients with Crohn’s Disease in Sustained Clinical Remission: A Lifetime Risk-Benefit Analysis
Source: PLoS One. 2016 Jun 6;11(6):e0157191. doi: 10.1371/journal.pone.0157191 (PMC4894633; doi:10.1371/journal.pone.0157191)

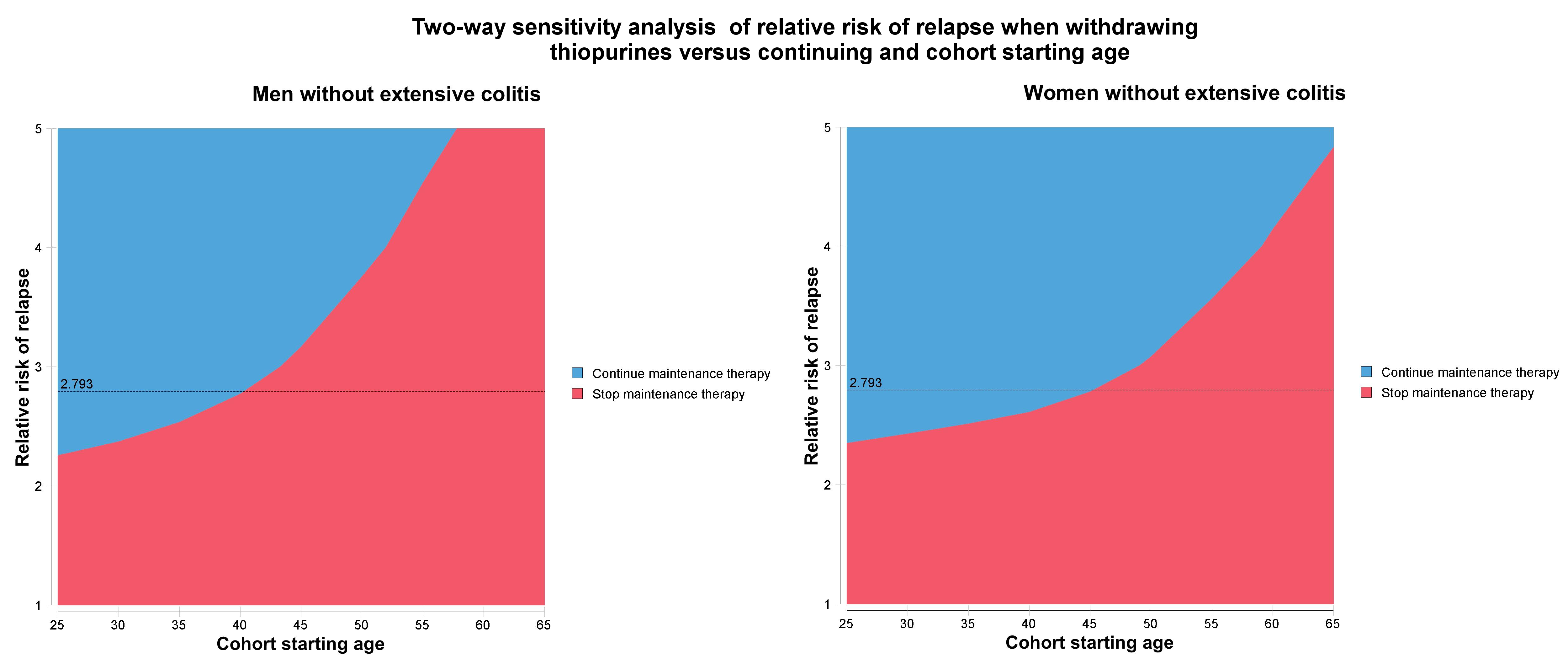

Supplement: S1 Fig — (TIFF) [file pone.0157191.s001.tiff]

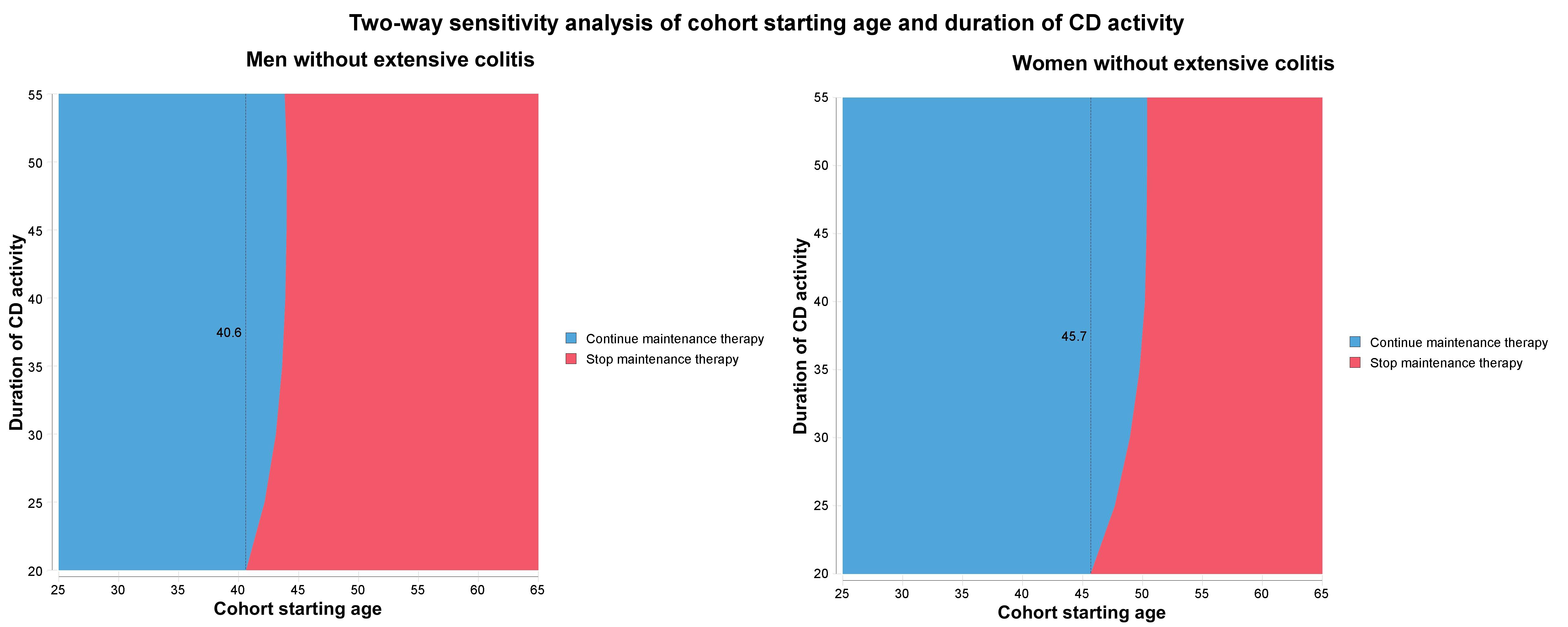

Supplement: S2 Fig — (TIFF) [file pone.0157191.s002.tiff]

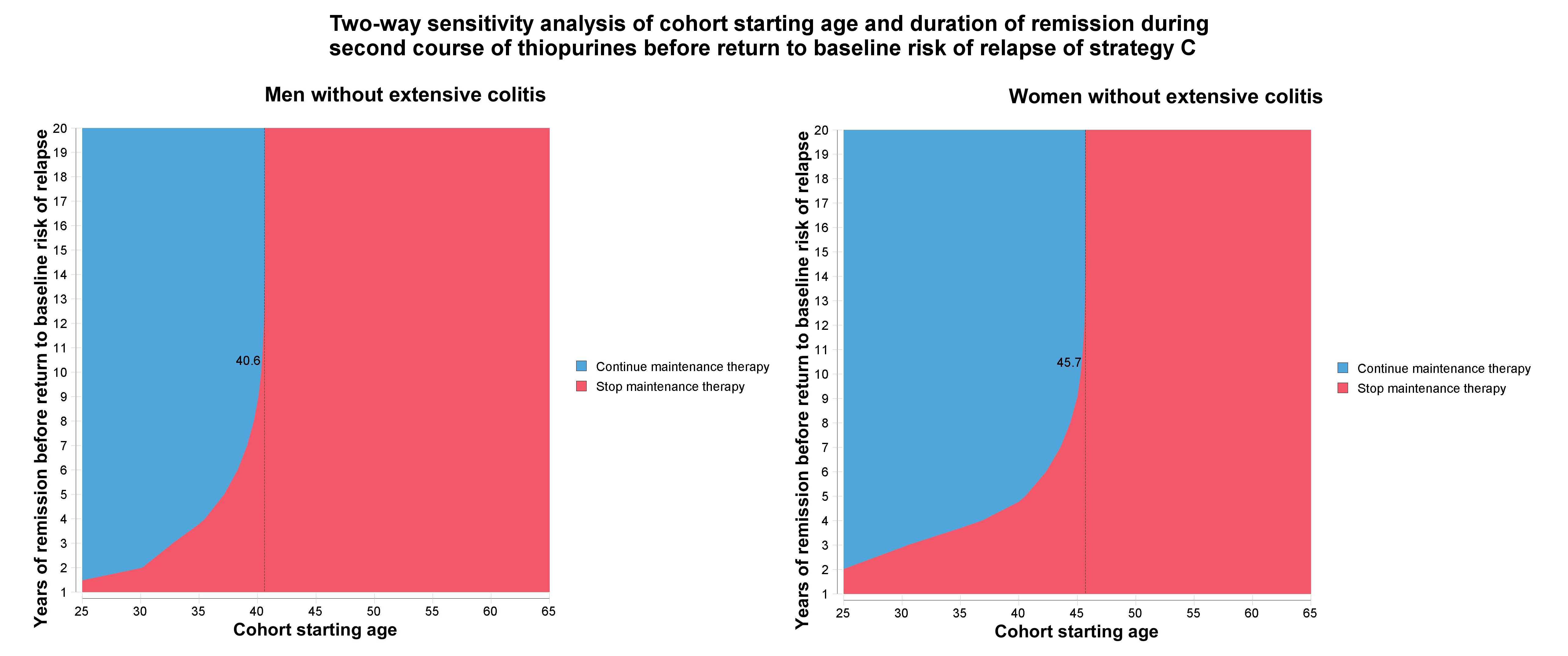

Supplement: S3 Fig — (TIFF) [file pone.0157191.s003.tiff]
