# Supplementary material for: Impact on Life Expectancy of Withdrawing Thiopurines in Patients with Crohn’s Disease in Sustained Clinical Remission: A Lifetime Risk-Benefit Analysis
Source: PLoS One. 2016 Jun 6;11(6):e0157191. doi: 10.1371/journal.pone.0157191 (PMC4894633; doi:10.1371/journal.pone.0157191)
Supplement: S1 Table — (DOC) [file pone.0157191.s004.doc]

| **Supplementary material. Table 1. Age- and gender-specific incidence rate of lymphoma, colorectal cancer, melanoma skin cancer, acute myeloid leukemia and bladder cancer, per 100,000 (2012 French National Cancer Registry)** | | | | | | | | | | | | |
| --- | --- | --- | --- | --- | --- | --- | --- | --- | --- | --- | --- | --- |
|  | **Lymphoma** | | **Colorectal  Cancer** | | **Melanoma  Skin cancer** | | **Non  MSC** | | **Urinary tract  cancer** | | **Acute myeloid  leukemia** | |
| Age category | Female | Male | Female | Male | Female | Male | Female | Male | Female | Male | Female | Male |
| 0-14 | 0.45 | 0.44 | 0.1 | 0.1 | 0.1 | 0.2 | 1.4 | 1.4 | 0.8 | 1 | 0.8 | 0.8 |
| 15-19 | 1.36 | 1.26 | 0.5 | 0.3 | 1 | 1.1 | 3 | 1.4 | 0.8 | 0.3 | 0.9 | 0.8 |
| 20-24 | 1.97 | 2.13 | 1.1 | 0.7 | 3.3 | 2.4 | 4.7 | 4.5 | 0.9 | 0.3 | 0.9 | 0.9 |
| 25-29 | 2.34 | 3.54 | 2 | 1.6 | 6.7 | 4.8 | 9.3 | 9.2 | 1.1 | 0.9 | 1 | 1.1 |
| 30-34 | 2.58 | 4.39 | 3.5 | 3.1 | 11.3 | 7.7 | 20.8 | 12,2 | 1.1 | 2.1 | 1.2 | 1.1 |
| 35-39 | 3.8 | 6.45 | 6.4 | 5.8 | 16.9 | 10.6 | 62 | 29 | 1.2 | 5.2 | 1.6 | 1.2 |
| 40-44 | 6.2 | 8.94 | 11.7 | 11.2 | 20.8 | 13.4 | 87 | 67.8 | 1.5 | 11.4 | 2.1 | 1.5 |
| 45-49 | 8.77 | 13.64 | 21.8 | 23.7 | 20.7 | 15.9 | 109.5 | 85.1 | 2.1 | 22.6 | 2.5 | 2.1 |
| 50-54 | 12.67 | 20.84 | 39.4 | 50.4 | 22.8 | 19.5 | 147.8 | 141.4 | 3.2 | 42.7 | 3.3 | 3.2 |
| 55-59 | 17.85 | 30.52 | 64.7 | 96.7 | 25.4 | 25.2 | 209.8 | 263.5 | 4.5 | 77 | 4.2 | 4.5 |
| 60-64 | 25.84 | 43.32 | 92.1 | 159.2 | 28.6 | 34.2 | 298.5 | 377.4 | 6.4 | 118.6 | 5.8 | 6.4 |
| 65-69 | 35.46 | 57.35 | 119.9 | 231.5 | 31.8 | 42.5 | 351 | 635.5 | 9.8 | 167.5 | 8.1 | 9.8 |
| 70-74 | 46.53 | 79.63 | 160.2 | 311.6 | 34.3 | 51.1 | 484.7 | 853.7 | 15.3 | 220.1 | 10.7 | 15.3 |
| 75-79 | 51.86 | 101.31 | 209.5 | 374.1 | 35.5 | 58.2 | 572.1 | 1180 | 20.9 | 264.5 | 12.9 | 20.9 |
| 80-84 | 56.92 | 109.15 | 260.3 | 438.2 | 38.9 | 68.2 | 723.3 | 1362 | 26 | 310 | 16.4 | 26 |
| 85-89 | 55.7 | 113.76 | 303.2 | 451.9 | 41.1 | 74 | 1060 | 1775 | 30.1 | 336.8 | 19.7 | 30.1 |
| 90-94 | 48.87 | 110.62 | 297.7 | 411.8 | 37.7 | 75.2 | 1060 | 1775 | 29.2 | 330.7 | 15.8 | 29.2 |
| 95+ | 40.49 | 105.5 | 226.6 | 327.2 | 37.6 | 72.7 | 1060 | 1775 | 24.2 | 307 | 12.5 | 24.2 |
|  |  |  |  |  |  |  |  |  |  |  |  |  |
