# Supplementary material for: Impact on Life Expectancy of Withdrawing Thiopurines in Patients with Crohn’s Disease in Sustained Clinical Remission: A Lifetime Risk-Benefit Analysis
Source: PLoS One. 2016 Jun 6;11(6):e0157191. doi: 10.1371/journal.pone.0157191 (PMC4894633; doi:10.1371/journal.pone.0157191)
Supplement: S2 Table — (DOC) [file pone.0157191.s005.doc]

| **Supplementary material. Table 2. Five-year relative mortality rates of cancer (2012 French National Cancer Registry)** | | | | | | | | | | | | | |
| --- | --- | --- | --- | --- | --- | --- | --- | --- | --- | --- | --- | --- | --- |
| **Colorectal Cancer** | |  |  |  |  |  |  | **Lymphoma** |  |  |  |  |  |
| Age category | Female | CI 95% | | Male | CI 95% | |  | Female | CI 95% | | Male | CI 95% | |
| 15-44 | 0.32 | 0.29 | 0.36 | 0.36 | 0.33 | 0.39 |  | 0.26 | 0.23 | 0.30 | 0.31 | 0.28 | 0.33 |
| 45-54 | 0.35 | 0.33 | 0.37 | 0.37 | 0.35 | 0.39 |  | 0.22 | 0.19 | 0.26 | 0.28 | 0.24 | 0.31 |
| 54-64 | 0.35 | 0.33 | 0.36 | 0.40 | 0.39 | 0.41 |  | 0.30 | 0.27 | 0.33 | 0.35 | 0.32 | 0.38 |
| 65-74 | 0.39 | 0.38 | 0.41 | 0.43 | 0.42 | 0.44 |  | 0.44 | 0.40 | 0.47 | 0.46 | 0.42 | 0.48 |
| 75+ | 0.50 | 0.49 | 0.51 | 0.51 | 0.49 | 0.52 |  | 0.64 | 0.61 | 0.67 | 0.65 | 0.61 | 0.68 |
| **Melanoma skin cancer** | |  |  |  |  |  |  | **Urinary tract cancer** | | |  |  |  |
| Age category | Female | CI 95% | | Male | CI 95% | |  | Female | CI 95% | | Male | CI 95% | |
| 15-44 | 0.06 | 0.04 | 0.07 | 0.14 | 0.11 | 0.16 |  | 0.22 | 0.16 | 0.28 | 0.24 | 0.19 | 0.29 |
| 45-54 | 0.10 | 0.07 | 0.12 | 0.15 | 0.12 | 0.18 |  | 0.26 | 0.21 | 0.31 | 0.30 | 0.27 | 0.33 |
| 54-64 | 0.10 | 0.07 | 0.12 | 0.13 | 0.10 | 0.16 |  | 0.31 | 0.28 | 0.36 | 0.34 | 0.33 | 0.36 |
| 65-74 | 0.15 | 0.12 | 0.18 | 0.18 | 0.15 | 0.22 |  | 0.39 | 0.35 | 0.42 | 0.41 | 0.39 | 0.43 |
| 75+ | 0.25 | 0.19 | 0.30 | 0.30 | 0.23 | 0.36 |  | 0.58 | 0.54 | 0.62 | 0.55 | 0.53 | 0.59 |
| **Acute myeloid leukemia** | |  |  |  |  |  |  |  |  |  |  |  |  |
| Age category | Female | CI 95% | | Male | CI 95% | |  |  |  |  |  |  |  |
| 15-44 | 0.46 | 0.4 | 0.52 | 0.57 | 0.5 | 0.63 |  |  |  |  |  |  |  |
| 45-54 | 0.67 | 0.59 | 0.75 | 0.64 | 0.56 | 0.7 |  |  |  |  |  |  |  |
| 54-64 | 0.72 | 0.64 | 0.78 | 0.74 | 0.68 | 0.79 |  |  |  |  |  |  |  |
| 65-74 | 0.88 | 0.84 | 0.91 | 0.90 | 0.87 | 0.93 |  |  |  |  |  |  |  |
| 75+ | 0.97 | 0.98 | 0.95 | 0.96 | 0.92 | 0.97 |  |  |  |  |  |  |  |
|  |  |  |  |  |  |  |  |  |  |  |  |  |  |
