# Supplementary material for: Impact on Life Expectancy of Withdrawing Thiopurines in Patients with Crohn’s Disease in Sustained Clinical Remission: A Lifetime Risk-Benefit Analysis
Source: PLoS One. 2016 Jun 6;11(6):e0157191. doi: 10.1371/journal.pone.0157191 (PMC4894633; doi:10.1371/journal.pone.0157191)
Supplement: S3 Table — (DOC) [file pone.0157191.s006.doc]

| **Supplementary material. Table 3. Age- and gender-specific mortality rate from other causes, per 100,000 (2010 French life tables)** | | |
| --- | --- | --- |
| Age category | Female | Male |
| 25-34 | 30.9 | 87.5 |
| 35-44 | 81.1 | 170.6 |
| 45-54 | 210.3 | 443.6 |
| 55-64 | 406.8 | 959.5 |
| 65-74 | 849.4 | 1838.1 |
| 75-84 | 2793.8 | 4891.6 |
| 85-94 | 9551 | 13467.6 |
| 95+ | 29789.4 | 35490.4 |
| * Excluding CD, Lymphoma, Melanoma and Colorectal Cancer. | | |
