# Supplementary material for: Impact on Life Expectancy of Withdrawing Thiopurines in Patients with Crohn’s Disease in Sustained Clinical Remission: A Lifetime Risk-Benefit Analysis
Source: PLoS One. 2016 Jun 6;11(6):e0157191. doi: 10.1371/journal.pone.0157191 (PMC4894633; doi:10.1371/journal.pone.0157191)
Supplement: S4 Table — (DOC) [file pone.0157191.s007.doc]

| **Supplementary material. Table 4. Model utility values.** | | | | | |
| --- | --- | --- | --- | --- | --- |
| **Model parameter** | | **Base** | **Min** | **Max** | **Source** |
| *Utility* |  |  |  |  |  |
|  | Severe flare medically treated | 0.620 | 0.434 | 0.806 | [7] |
|  | Severe flare with surgery | 0.400 | 0.280 | 0.520 | [8] |
|  | Moderate flare | 0.740 | 0.518 | 1.000 | [7] |
|  | Remission | 0.880 | 0.616 | 1.000 | [7] |
|  | Adverse event (neutropenia, SAE due to anti-TNF) | 0.620 | 0.434 | 0.806 | [9] |
|  | Lymphoma | 0.470 | 0.329 | 0.611 | [10] |
|  | Colorectal cancer | 0.694 | 0.486 | 0.902 | [11] |
|  | Melanoma skin cancer | 0.720 | 0.504 | 0.936 | [12] |
|  | Non melanoma skin cancer | 0.990 | 0.693 | 1.000 | [13] |
|  | | | | | |
