# Supplementary material for: Impact on Life Expectancy of Withdrawing Thiopurines in Patients with Crohn’s Disease in Sustained Clinical Remission: A Lifetime Risk-Benefit Analysis
Source: PLoS One. 2016 Jun 6;11(6):e0157191. doi: 10.1371/journal.pone.0157191 (PMC4894633; doi:10.1371/journal.pone.0157191)
Supplement: S5 Table — (DOC) [file pone.0157191.s008.doc]

| **Supplementary material. Table 5 .Model parameter distribution** | | | | | | |
| --- | --- | --- | --- | --- | --- | --- |
| **Model parameter** | | | **Distribution** |  |  |  |
| *Target population characteristics* | | |  |  |  |  |
|  | Female (%) | | if Uniform(0,1)>0,6 |  |  |  |
|  | Duration of Crohn's disease activity, years | | Uniform (minimum, maximum) | 0 | 30 |  |
| *Annual risk of relapse* | | |  |  |  |  |
|  | *Before intestinal resection* | |  |  |  |  |
|  |  | Thiopurines withdrawal | Beta (alpha, beta) | 15 | 43 |  |
|  |  | Thiopurines continuation | Beta (alpha, beta) | 5 | 49 |  |
|  |  | Thiopurines resumed after withdrawal | Beta (alpha, beta) | 24 | 43 |  |
|  |  | Thiopurines and Infliximab | Beta (alpha, beta) | 92 | 173 |  |
|  |  | Infliximab monotherapy | Beta (alpha, beta) | 171 | 148 |  |
|  |  | Infliximab optimized and thiopurines | Beta (alpha, beta) | 58 | 54 |  |
|  |  | Infliximab optimized monotherapy | Beta (alpha, beta) | 25 | 22 |  |
|  |  | Primary non-response to Infliximab | Beta (alpha, beta) | 43 | 426 |  |
|  |  | Thiopurines and Adalimumab | Beta (alpha, beta) | 51 | 39 |  |
|  |  | Adalimumab monotherapy | Beta (alpha, beta) | 271 | 201 |  |
|  |  | Adalimumab after optimization  (with or without thiopurines) | Beta (alpha, beta) | 19 | 17 |  |
|  |  | Primary non-response to Adalimumab | Beta (alpha, beta) | 18 | 420 |  |
|  |  | Steroids only for induction of remission | Beta (alpha, beta) | 53 | 79 |  |
|  | *After intestinal resection* | | Beta (alpha, beta) | 37 | 94 |  |
|  |  | Thiopurines | Beta (alpha, beta) | 31 | 185 |  |
|  |  | Infliximab (without or with thiopurines) | Beta (alpha, beta) | 1 | 24 |  |
|  |  | Adalimumab (without or with thiopurines) | Beta (alpha, beta) | 1 | 15 |  |
| *Relapse-related events* | | |  |  |  |  |
|  | Severe relapse | | Beta (alpha, beta) | 104 | 253 |  |
|  | Surgery for severe relapse | | Beta (alpha, beta) | 40 | 64 |  |
|  | Intestinal resection for surgery | | Beta (alpha, beta) | 110 | 42 |  |
|  | Duration of decreased risk of relapse after Intestinal resection (years) | | Uniform (minimum, maximum) | 0 | 5 |  |
| *Annual risk of Serious Adverse Events* | | |  |  |  |  |
|  | Opportunistic (viral) infection with thiopurines | | Beta (alpha, beta) | 70 | 40509 |  |
|  | Opportunistic infection with anti-TNF drugs | | Beta (alpha, beta) | 87 | 7622 |  |
|  | SAE due to infliximab (opportunistic infections and cancers excluded) | | Uniform (minimum, maximum) | 0.013 | 0.022 |  |
|  | SAE due to adalimumab (opportunistic infections and cancers excluded) | | Uniform (minimum, maximum) | 0.001 | 0.003 |  |
|  | Standardized Incidence Ratio of Lymphoma | |  |  |  |  |
|  |  | Thiopurines continuation | Triangular (likiest, min, max) | 4.92 | 3.10 | 7.78 |
|  | Standardized Incidence Ratio of Colorectal cancer | |  |  |  |  |
|  |  | Longstanding colitis | Triangular (likiest, min, max) | 9.04 | 2.58 | 15.5 |
|  |  | Longstanding colitis, Thiopurines continuation | Triangular (likiest, min, max) | 0.28 | 0.01 | 0.55 |
|  | Standardized Incidence Ratio of melanoma skin cancer(MSC) | |  |  |  |  |
|  |  | Anti-TNF (any use) | Triangular (likiest, min, max) | 1.37 | 1.04 | 1.70 |
|  | Standardized Incidence Ratio of non-melanoma skin cancer | |  |  |  |  |
|  |  | Thiopurines (any use) | Triangular (likiest, min, max) | 2.28 | 1.11 | 3.45 |
| *Mortality rates of Severe Adverse Events* | | |  |  |  |  |
|  | Opportunistic infection with thiopurines | | Beta (alpha, beta) | 2 | 68 |  |
|  | Opportunistic infection with anti-TNF drugs | | Beta (alpha, beta) | 4 | 83 |  |
|  | Age-adjusted mortality rate of severe flare without surgery | | Triangular (likiest, min, max) | 0.0021 | 0.0006 |  |
|  | Age-adjusted mortality rate of severe flare with surgery | | Triangular (likiest, min, max) | 0.0103 | 0.0029 |  |
|  |  |  |  |  |  |  |
