# Supplementary material for: Impact on Life Expectancy of Withdrawing Thiopurines in Patients with Crohn’s Disease in Sustained Clinical Remission: A Lifetime Risk-Benefit Analysis
Source: PLoS One. 2016 Jun 6;11(6):e0157191. doi: 10.1371/journal.pone.0157191 (PMC4894633; doi:10.1371/journal.pone.0157191)
Supplement: S6 Table — (DOC) [file pone.0157191.s009.doc]

| **Supplementary material. Table 6. Life expectancy and causes of death associated with continuing (C) or withdrawing (W) from maintenance therapy with thiopurines in stratified cohorts** | | | | | | | | | | | | | | | | | | | | |
| --- | --- | --- | --- | --- | --- | --- | --- | --- | --- | --- | --- | --- | --- | --- | --- | --- | --- | --- | --- | --- |
|  |  | **Male, 35y.,  CD still active for 15y.** | | **Male, 35y.,  CD still active for 15y. Involving EC** | | | **Male, 65y.,  CD still active for 15y.** | | | | **Male, 65y.,  CD still active for 15y. Involving EC** | | **Female, 35y.,  CD still active for 15y.** | | **Female, 35y.,  CD still active for 15y. Involving EC** | | **Female, 65y.,  CD still active for 15y.** | | **Female, 65y.,  CD still active for 15y. Involving EC** | |
|  |  | **C** | **W** | **C** | **W** | | | **C** | **W** | | **C** | **W** | **C** | **W** | **C** | **W** | **C** | **W** | **C** | **W** |
| Life expectancy, age at death | | 73.99 | 73.97 | 73.95 | | 73.87 | | 79.54 | | 79.61 | 79.37 | 79.28 | 79.86 | 79.83 | 79.82 | 79.74 | 82.97 | 83.02 | 82.85 | 82.75 |
| Loss in life expectancy as compared to general population, years | | -0.25 | -0.27 | -0.28 | | -0.36 | | -0.18 | | -0.11 | -0.34 | -0.45 | -0.28 | -0.31 | -0.32 | -0.40 | -0.17 | -0.12 | -0.29 | -0.39 |
| Quality-adjusted life expectancy, age at death | | 68.48 | 67.75 | 68.44 | | 67.66 | | 77.04 | | 76.68 | 76.88 | 76.35 | 73.63 | 72.90 | 73.60 | 72.81 | 80.04 | 79.54 | 79.93 | 79.27 |
| Causes of death, % | |  |  |  | |  | |  | |  |  |  |  |  |  |  |  |  |  |  |
|  | Severe relapse without surgery | 0.08% | 0.16% | 0.08% | | 0.16% | | 0.07% | | 0.12% | 0.07% | 0.12% | 0.08% | 0.16% | 0.08% | 0.16% | 0.07% | 0.14% | 0.07% | 0.14% |
|  | Severe relapse  with surgery | 0.23% | 0.48% | 0.23% | | 0.48% | | 0.21% | | 0.37% | 0.20% | 0.36% | 0.24% | 0.49% | 0.24% | 0.49% | 0.22% | 0.42% | 0.22% | 0.41% |
|  | Opportunistic infection | 0.18% | 0.12% | 0.18% | | 0.12% | | 0.17% | | 0.09% | 0.16% | 0.08% | 0.19% | 0.12% | 0.19% | 0.12% | 0.18% | 0.10% | 0.17% | 0.10% |
|  | Lymphoma | 0.76% | 0.67% | 0.76% | | 0.67% | | 1.81% | | 1.01% | 1.78% | 0.96% | 0.53% | 0.48% | 0.53% | 0.48% | 1.28% | 0.72% | 1.27% | 0.70% |
|  | Colorectal cancer | 1.90% | 1.91% | 2.03% | | 2.25% | | 1.61% | | 1.63% | 3.69% | 5.63% | 1.49% | 1.50% | 1.61% | 1.79% | 1.21% | 1.22% | 2.46% | 3.96% |
|  | Melanoma skin cancer | 0.57% | 0.21% | 0.57% | | 0.21% | | 0.31% | | 0.17% | 0.30% | 0.16% | 0.63% | 0.18% | 0.63% | 0.18% | 0.24% | 0.12% | 0.24% | 0.12% |
|  | Other causes of death | 96.28% | 96.45% | 96.15% | | 96.11% | | 95.82% | | 96.61% | 93.80% | 92.69% | 96.84% | 97.07% | 96.72% | 96.78% | 96.80% | 97.28% | 95.57% | 94.57% |
|  | Relapse | 0.31% | 0.64% | 0.31% | | 0.64% | | 0.28% | | 0.49% | 0.27% | 0.48% | 0.32% | 0.65% | 0.32% | 0.65% | 0.29% | 0.56% | 0.29% | 0.55% |
|  | SAE | 3.41% | 2.91% | 3.54% | | 3.25% | | 3.90% | | 2.90% | 5.93% | 6.83% | 2.84% | 2.28% | 2.96% | 2.57% | 2.91% | 2.16% | 4.14% | 4.88% |
