# Supplementary material for: Impact on Life Expectancy of Withdrawing Thiopurines in Patients with Crohn’s Disease in Sustained Clinical Remission: A Lifetime Risk-Benefit Analysis
Source: PLoS One. 2016 Jun 6;11(6):e0157191. doi: 10.1371/journal.pone.0157191 (PMC4894633; doi:10.1371/journal.pone.0157191)
Supplement: S7 Table — (DOC) [file pone.0157191.s010.doc]

| **Supplementary material. Table 7 Events associated with continuing (C) or withdrawing (W) from maintenance therapy with thiopurines** | | | | | | | | | | | | | | | | | |
| --- | --- | --- | --- | --- | --- | --- | --- | --- | --- | --- | --- | --- | --- | --- | --- | --- | --- |
|  |  | **Male, 35y.,  CD still active for 15y.** | | **Male, 35y.,  CD still active for 15y. Involving EC** | | **Male, 65y.,  CD still active for 15y.** | | **Male, 65y.,  CD still active for 15y. Involving EC** | | **Female, 35y.,  CD still active for 15y.** | | **Female, 35y.,  CD still active for 15y. Involving EC** | | **Female, 65y.,  CD still active for 15y.** | | **Female, 65y.,  CD still active for 15y. Involving EC** | |
|  |  | **C** | **W** | **C** | **W** | **C** | **W** | **C** | **W** | **C** | **W** | **C** | **W** | **C** | **W** | **C** | **W** |
| Events for 1,000 patients-years | |  |  |  |  |  |  |  |  |  |  |  |  |  |  |  |  |
|  | Severe relapse (during CD activity) | 40.64 | 84.99 | 40.62 | 84.94 | 46.05 | 80.68 | 44.89 | 79.89 | 41.15 | 85.25 | 41.13 | 85.21 | 43.85 | 82.67 | 43.38 | 82.25 |
|  | Lymphoma | 0.58 | 0.49 | 0.58 | 0.49 | 0.99 | 0.56 | 0.98 | 0.54 | 0.36 | 0.31 | 0.36 | 0.31 | 0.63 | 0.36 | 0.62 | 0.35 |
|  | Colorectal cancer | 1.47 | 1.48 | 1.57 | 1.75 | 1.10 | 1.11 | 2.46 | 3.71 | 1.02 | 1.03 | 1.11 | 1.25 | 0.76 | 0.77 | 1.49 | 2.35 |
|  | Opportunistic infection (during drug exposure) | 3.31 | 1.93 | 3.31 | 1.93 | 3.63 | 1.71 | 3.54 | 1.68 | 3.35 | 1.95 | 3.35 | 1.95 | 3.50 | 1.81 | 3.46 | 1.80 |
| Relative risk between continuation and withdrawal strategy | |  |  |  |  |  |  |  |  |  |  |  |  |  |  |  |  |
|  | Severe relapse | 0.48 |  | 0.48 |  | 0.57 |  | 0.56 |  | 0.48 |  | 0.48 |  | 0.53 |  | 0.53 |  |
|  | Lymphoma | 1.18 |  | 1.18 |  | 1.77 |  | 1.83 |  | 1.16 |  | 1.16 |  | 1.77 |  | 1.79 |  |
|  | Colorectal cancer | 1.00 |  | 0.90 |  | 0.99 |  | 0.66 |  | 0.99 |  | 0.89 |  | 0.99 |  | 0.63 |  |
|  | Opportunistic infection | 1.72 |  | 1.72 |  | 2.12 |  | 2.11 |  | 1.72 |  | 1.72 |  | 1.93 |  | 1.92 |  |
| Relative risk compared to general population | |  |  |  |  |  |  |  |  |  |  |  |  |  |  |  |  |
|  | Lymphoma | 1.39 | 1.18 | 1.39 | 1.17 | 3.44 | 1.94 | 10.48 | 5.76 | 1.35 | 1.16 | 1.35 | 1.16 | 3.45 | 1.95 | 9.17 | 5.14 |
|  | Colorectal cancer | 0.99 | 1.00 | 1.06 | 1.19 | 0.98 | 0.99 | 6.79 | 10.27 | 0.99 | 1.00 | 1.07 | 1.21 | 0.99 | 0.99 | 5.16 | 8.19 |
|  |  |  |  |  |  |  |  |  |  |  |  |  |  |  |  |  |  |
