# Supplementary material for: Impact on Life Expectancy of Withdrawing Thiopurines in Patients with Crohn’s Disease in Sustained Clinical Remission: A Lifetime Risk-Benefit Analysis
Source: PLoS One. 2016 Jun 6;11(6):e0157191. doi: 10.1371/journal.pone.0157191 (PMC4894633; doi:10.1371/journal.pone.0157191)
Supplement: S1 Text — (DOC) [file pone.0157191.s011.doc]

# Supplementary material

## Complementary sensitivity analysis

### Inclusion of acute myeloid leukemia and urinal tract cancer

SIR of acute myeloid leukemia and urinal tract cancer were derivated from the CESAME cohort. The age threshold beyond which withdrawing thiopurines became the preferred strategy decreased to 31.9 years for men (base-case 40.6) and 33 years for women (base-case 45.7) without EC. In case of EC, strategy W was preferred at 58.4 years for men and strategy C remained the preferred strategy for women regardless of cohort starting age.

### Inclusion of bone marrow suppression risk

Immunosuppressive treatment with thiopurines carries risks due to myelotoxicity, although most severe neutropenia occurs within the first year of treatment.[1,2] We considered in a sensitivity analysis an excess risk of bone marrow suppression for patients under thiopurines. An episode of bone marrow suppression leads to withdrawal of thiopurines and monotherapy of anti-TNFs if required. Annual risks were estimated from the French National Hospital DRG database (PMSI). We found 61 incident cases of severe neutropenia in 2008-2009 for patients having not received anti-TNF therapies in an outpatient setting. Drug exposure was estimated indirectly with use of the proportion of CD patients treated in the CESAME cohort. Accordingly, we calculated that the incidence rate of severe neutropenia in CD patients aged 25 to 80 years old and treated by thiopurines was 1.5 (CI 95%, 1.2; 1.9) per 1,000 patient-years. Our figure was below the range of the published estimate from a recent meta-analysis (3%; CI95%, 3; 4%).[1] Two patients out of 61 patients died within 3 months of the severe neutropenia onset (3.3%; CI 95%, 0.4; 11.4%), and our risk of death did not differ significantly from published estimates.[1]

In the alternative model including an excess risk of bone marrow suppression under thiopurines, the age threshold at which withdrawal strategy becomes the preferred strategy above decreased to 36.1 years for men (base case 40.6) and 37.4 years for women (base-case 45.7) without EC. In case of EC, continuation strategy remained the preferred strategy regardless of age.

## Model validation

### Internal validity

We assumed that disease activity lasts for 20 years and thereafter patients resumed to the life expectancy of the general population. This assumption is based on the fact that natural history of CD is unpredictable[3] and new drugs will potentially change the disease evolution in the next 20 years.[4,5] Considering a constant annual risk of relapse during whole life may overestimate the severity of the disease. However, the decision was not modified in the two-way sensitivity analysis on duration of CD activity from 20 to 55 years. The study results were robust to longer duration of CD activity for all stratified cohorts (Supplementary material Figure 2). When the duration of CD activity was increased from 20 (base-case) to 55 years (lifetime), withdrawing thiopurines became the preferred strategy only slightly later in patients without extensive colitis (41.7 to 45 years in men; 46.9 to 51.6 in women); continuing thiopurines (C) remained the preferred strategy in patients with extensive colitis.

If patients back on maintenance therapy with thiopurines resumed to the baseline risk of relapse of strategy C after 5 years of remission, strategy W became the preferred strategy after 37.2 years in men and 38.8 years in women. If patients back on maintenance therapy with thiopurines resumed to the baseline risk of relapse of strategy C after 1 year of remission, strategy W was preferred regardless of age (Supplementary material Figure 3).

We used the hazard ratio for advanced neoplasia between patients who received thiopurines compared with those who never received thiopurines from the CESAME cohort as a proxy for hazard ratio for colorectal cancer between patients under thiopurines and patients without thiopurines. However, the decision to continue thiopurines in case of extensive colitis remained the preferred strategy in all of our sensitivity analysis for 35-year old patients, including on variation of hazard ratio and relative risk of colorectal cancer in case of extensive colitis between their 95% confidence bounds.

### External validity

In order to validate our base-case model, we compared the simulated cumulative incidence rate of life-threatening events to that observed in epidemiological studies. The cumulate 20 year incidence rate of surgery was between 19% and 47% in all stratified cohorts. This rate is comparable to the 20-year incidence rate ranged from 47.4 % (CI 95%, 26.4; 65.2%) to 69.7% (CI 95%, 48.2; 82.3%) observed in the Olmsted county cohort,[6] considering that this study suggests that the rate of surgery in the first 5 years post-diagnosis is higher than in subsequent years of the clinical course rate of surgery and considering that our cohort begins after 5 years of disease onset. Furthermore, the annual risk of surgery was estimated from a subset of CD patient of the MICISTA cohort in sustained clinical remission of thiopurines.

# References

1. Gisbert JP, Gomolln F. Thiopurine-Induced Myelotoxicity in Patients With Inflammatory Bowel Disease: A Review. Am J Gastroenterol. 2008;103: 1783–1800. doi:10.1111/j.1572-0241.2008.01848.x

2. Lewis JD, Abramson O, Pascua M, Liu L, Asakura LM, Velayos FS, et al. Timing of Myelosuppression During Thiopurine Therapy for Inflammatory Bowel Disease: Implications for Monitoring Recommendations. Clin Gastroenterol Hepatol. 2009;7: 1195–1201. doi:10.1016/j.cgh.2009.07.019

3. Cosnes J, Gower-Rousseau C, Seksik P, Cortot A. Epidemiology and natural history of inflammatory bowel diseases. Gastroenterology. 2011;140: 1785–1794. doi:10.1053/j.gastro.2011.01.055

4. Sandborn WJ, Gasink C, Gao L-L, Blank MA, Johanns J, Guzzo C, et al. Ustekinumab Induction and Maintenance Therapy in Refractory Crohn’s Disease. N Engl J Med. 2012;367: 1519–1528. doi:10.1056/NEJMoa1203572

5. Sandborn WJ, Feagan BG, Rutgeerts P, Hanauer S, Colombel J-F, Sands BE, et al. Vedolizumab as Induction and Maintenance Therapy for Crohn’s Disease. N Engl J Med. 2013;369: 711–721. doi:10.1056/NEJMoa1215739

6. Peyrin-Biroulet L, Harmsen WS, Tremaine WJ, Zinsmeister AR, Sandborn WJ, Loftus EV Jr. Surgery in a population-based cohort of Crohn’s disease from Olmsted County, Minnesota (1970-2004). Am J Gastroenterol. 2012;107: 1693–1701. doi:10.1038/ajg.2012.298

7. Gregor JC, McDonald JW, Klar N, Wall R, Atkinson K, Lamba B, et al. An evaluation of utility measurement in Crohn’s disease. Inflamm Bowel Dis. 1997;3: 265–276.

8. Lindsay J, Punekar YS, Morris J, Chung-Faye G. Health-economic analysis: cost-effectiveness of scheduled maintenance treatment with infliximab for Crohn’s disease--modelling outcomes in active luminal and fistulizing disease in adults. Aliment Pharmacol Ther. 2008;28: 76–87. doi:10.1111/j.1365-2036.2008.03709.x

9. Scott FI, Vajravelu RK, Bewtra M, Mamtani R, Lee D, Goldberg DS, et al. The benefit-to-risk balance of combining infliximab with azathioprine varies with age: a markov model. Clin Gastroenterol Hepatol. 2015;13: 302–309.e11. doi:10.1016/j.cgh.2014.07.058

10. Lewis JD, Schwartz JS, Lichtenstein GR. Azathioprine for maintenance of remission in Crohn’s disease: benefits outweigh the risk of lymphoma. Gastroenterology. 2000;118: 1018–1024.

11. Nguyen GC, Frick KD, Dassopoulos T. Medical decision analysis for the management of unifocal, flat, low-grade dysplasia in ulcerative colitis. Gastrointest Endosc. 2009;69: 1299–1310. doi:10.1016/j.gie.2008.08.042

12. Tromme I, Devleesschauwer B, Beutels P, Richez P, Leroy A, Baurain J-F, et al. Health-related quality of life in patients with melanoma expressed as utilities and disability weights. Br J Dermatol. 2014;171: 1443–1450. doi:10.1111/bjd.13262

13. Lear W, Akeroyd JE, Mittmann N, Murray C. Measurement of utility in nonmelanoma skin cancer. J Cutan Med Surg. 2008;12: 102–106.
